# Obesity, Polycystic Ovary Syndrome, and Infertility: A New Avenue for GLP-1 Receptor Agonists

**DOI:** 10.1210/clinem/dgaa285

**Published:** 2020-05-22

**Authors:** Hellas Cena, Luca Chiovato, Rossella E Nappi

**Affiliations:** 1 Laboratory of Dietetics and Clinical Nutrition, Department of Public Health, Experimental and Forensic Medicine, University of Pavia, Pavia, Italy; 2 Clinical Nutrition and Dietetics Service, Unit of Internal Medicine and Endocrinology, Istituti Clinici Scientifici Maugeri IRCCS, Pavia, Italy; 3 Unit of Internal Medicine and Endocrinology, Laboratory for Endocrine Disruptors, Istituti Clinici Scientifici Maugeri IRCCS, Pavia, Italy; 4 Department of Internal Medicine and Therapeutics, University of Pavia, Pavia, Italy; 5 Research Center for Reproductive Medicine, Gynecological Endocrinology and Menopause, IRCCS San Matteo Foundation, Pavia, Italy; 6 Department of Clinical, Surgical, Diagnostic and Pediatric Sciences, University of Pavia, Pavia, Italy

**Keywords:** obesity, infertility, polycystic ovary syndrome, GLP-1 receptor agonists

## Abstract

**Context:**

Obesity is responsible for an increased risk of sub-fecundity and infertility. Obese women show poorer reproductive outcomes regardless of the mode of conception, and higher body mass index (BMI) is associated with poorer fertility prognosis. Polycystic ovary syndrome (PCOS) is one of the leading causes of infertility, and many women with PCOS are also overweight or obese.

**Evidence Acquisition:**

The aim of the present narrative review is to describe the mechanisms responsible for the development of infertility and PCOS in women with obesity/overweight, with a focus on the emerging role of glucagon-like peptide-1 (GLP-1) receptor agonists (GLP-1 RAs) as a therapeutic option for obese women with PCOS.

**Evidence synthesis:**

Weight reduction represents the most significant factor affecting fertility and pregnancy outcomes. Current experimental and clinical evidence suggests the presence of an underlying pathophysiological link between obesity, GLP-1 kinetic alterations, and PCOS pathogenesis. Based on the positive results in patients affected by obesity, with or without diabetes, the administration of GLP-1 RA (mainly liraglutide) alone or in combination with metformin has been investigated in women with obesity and PCOS. Several studies demonstrated significant weight loss and testosterone reduction, with mixed results relative to improvements in insulin resistance parameters and menstrual patterns.

**Conclusions:**

The weight loss effects of GLP-1 RA offer a unique opportunity to expand the treatment options available to PCOS patients.

Obesity represents a global pandemic, with profound clinical, social, and economic consequences, both in developed countries and in developing nations. Worldwide, the prevalence of obesity has nearly tripled since 1975, and more than 1.9 billion adults were overweight in 2016, of whom over 650 million were obese (1). Obesity is one of the leading causes of premature death and its consequences are not limited to the adult population; indeed, 41 million children under the age of 5 and over 340 million children and adolescents aged 5 to 19 were overweight or obese in 2016 (1). Given these trends, obesity *per se* shortens the life and health expectancy of the current generation of children in comparison with earlier generations (2).

Women of reproductive age are not spared from this dramatic trend; in the United States, 34% of women in this age group are obese (3). Obesity is responsible for an increased risk of subfecundity and infertility, mainly related to impairment in the hypothalamic-pituitary-ovarian (HPO) axis, poor oocyte quality, and altered endometrial receptivity (4, 5). Obese women show poorer reproductive outcomes regardless of the mode of conception, and higher body mass index (BMI) is associated with poorer fertility prognosis (6). Polycystic ovary syndrome (PCOS) is one of the leading causes of infertility, being the most common endocrine disorder in women of reproductive age. Diagnostic criteria include hyperandrogenism, oligo-anovulation, and polycystic ovary morphology (7), but many women with PCOS are also overweight or obese (8). Adiposity has a strong influence in distinct PCOS phenotypes and affects the management of symptoms and fertility outcomes (9).

The aim of the present narrative review is to describe the mechanisms responsible for the development of infertility and PCOS in women with obesity/overweight, with a focus on the emerging role of glucagon-like peptide-1 (GLP-1) receptor agonists (GLP-1 RA) as a therapeutic option for obese women with PCOS.

## Obesity and Fertility in Women

Obesity, a key component of metabolic syndrome, exerts a significant impact on female fertility, primarily due to functional alteration of the HPO axis. Obesity is frequently associated with higher circulating levels of insulin, with subsequent increased ovarian androgen production (10). The excess adipose tissue is responsible for the aromatization of these androgens to estrogen, leading to a negative feedback on the HPO axis and affecting gonadotropin production (11). These alterations are responsible for ovulatory dysfunction and menstrual abnormalities.

Hyperinsulinemia plays a fundamental role in the pathogenesis of PCOS, characterized by oligomenorrhea and hyperandrogenism. The concomitant presence of obesity further increases insulin resistance and exacerbates the symptoms of PCOS (12). On the other hand, the increased androgen production in PCOS causes deposition of visceral fat, which in turn accentuates insulin resistance and hyperinsulinemia, further fueling this vicious cycle (13).

Several studies have shown that obesity is associated with increased time-to-pregnancy. An inverse relationship between increasing BMI and fecundability ratios was demonstrated in 2 large cohorts of Danish women planning pregnancies (4, 14). Of note, subfertility in obese women is present even in the absence of ovulatory dysfunction. Reduced fecundity in eumenorrheic obese women was documented in a large US cohort of more than 7000 women (15). In a Dutch cohort of more than 3000 women with normal cycles, the likelihood of spontaneous conception declined linearly with BMI over 29 kg/m^2^. Corrected for possible related factors, women with high BMI had a 4% lower pregnancy rate per BMI unit increase (5).

Several lines of evidence support a negative impact of obesity also on assisted reproduction techniques (ART) outcomes. Indeed, obesity may prolong the duration of ovulation induction, increase the gonadotrophin dose, decrease the number of mature follicles and oocytes retrieved, and increase the cycle cancellation rate (16). Moreover, obesity may have a negative impact on oocyte and embryo quality. Therefore, fertilization, embryo transfer, implantation, and pregnancy rates have been frequently reported to be low correlating to BMI categories (6, 13). In addition, obesity can make oocyte retrieval and embryo transfer procedures more difficult (6). Finally, several studies indicated an increased risk of miscarriage in obese women in ART cycles. A meta-analysis of these studies showed that women with BMI ≥25 kg/m^2^ had significantly higher odds of miscarriage, regardless of the conception method (OR 1.67; 95% CI, 1.25-2.25) (17).

The notion that the extremes of maternal BMI may decrease success rates of fertility interventions and increase maternal-fetal morbidity has prompted many providers to establish BMI cutoffs for fertility treatment (18, 19).

### Metabolic syndrome and infertility

Obesity is frequently associated with glucose intolerance, insulin resistance, dyslipidemia, and hypertension. The clustering of these risk factors is usually referred to as metabolic syndrome (20). Many of the abnormalities of metabolic syndrome overlap with those of PCOS, and it is hypothesized that both conditions might share the same pathogenesis, namely, hyperinsulinemia and glucose intolerance (20). A link between PCOS and metabolic syndrome has been documented in several studies (21-23), with a prevalence of metabolic syndrome among women with PCOS ranging between 33% and 46%.

In addition to the negative impact of obesity and insulin resistance on female fertility, metabolic disorders can directly or indirectly affect the fertility of women by influencing either pituitary-hypothalamic function or ovarian function. In particular, both type 1 and type 2 diabetes mellitus (DM) have been associated with undesirable effects on the women’s reproductive axis. Individuals with DM and primary and secondary amenorrhea exhibit low levels of estradiol (24), luteinizing hormone (LH), and follicle stimulating hormone (FSH), which have mostly been associated with a lack of residual insulin secretion (25) and poor metabolic control (26). Furthermore, the “glucotoxic” effect of chronic hyperglycemia on the neurons of the hypothalamus can be responsible for reduced LH response to gonadotropin-releasing hormone (GnRH) stimuli (27).

Hyperglycemia also affects ovarian function in women. Elevated blood glucose levels trigger peripheral insulin resistance. In addition, hyperglycemia can also affect ovarian function via the accumulation of advanced glycation products (27, 28). Collectively, nutrients, especially glucose availability, are crucial metabolic regulators of reproductive function (29, 30).

Type 1 DM is associated with fewer pregnancies and live births (31). However, studies assessing fertility treatments in women with diabetes show that achieving optimal metabolic control is the key factor in ensuring successful pregnancy (24). In addition, in vitro fertilization treatments in women with type 1 DM and optimal metabolic control produce similar outcomes compared to those in nondiabetic women (26).

In addition to insulin resistance and hyperglycemia, also dyslipidemia, typical component of metabolic syndrome, has been suggested to have negative effects on fertility and pregnancy (32). A recent meta-analysis documented that infertile women had statistically significant higher BMI, increased total cholesterol, low-density lipoprotein (LDL)-cholesterol, and triglycerides compared to fertile women (33). A subgroup analysis revealed that total cholesterol, high-density lipoprotein (HDL)-cholesterol, fasting glucose, and fasting insulin were increased only in women with PCOS compared with fertile women, while BMI, triglycerides, and LDL-cholesterol were significantly increased in women with any cause of infertility relative to fertile women. However, the causal relationship between dyslipidemia and infertility remains to be proven.

### Weight reduction as a treatment target in women with PCOS or infertility

Considering the impact of obesity and metabolic disorders on fertility, it seems reasonable to assume that weight reduction would provide benefit. Existing literature on the effect of weight loss in obese women desiring conception is mixed. In a study on 67 obese anovulatory infertile women undergoing a 6-month weight loss program, participants lost an average of 10 kg, and ovulatory function was restored in 60 women (90%), of whom 52 (78%) conceived with a miscarriage rate of 18% (34).

In a cohort of 170 women undergoing in vitro fertilization (IVF), short-term weight loss was associated with a higher proportion of retrieved metaphase II oocytes (35). This association was stronger among women with overweight or obesity at baseline. However, short-term weight loss was not correlated with positive β-hCG test, clinical pregnancy, or live birth rates (LBRs) (35). Another retrospective cohort study involved 52 overweight or obese women with infertility who were referred to weight loss counseling. Patients achieving the goal of 10% weight loss had significantly higher conception rates and LBRs (36).

In a trial involving 49 women with obesity who were undergoing fertility treatment, participants were randomized to an intensive 12-week lifestyle intervention or to a control group. Women allocated to the intervention group achieved an average 6.6 kg weight loss and a significantly higher LBR than the control group (44% vs 14%); they also required fewer treatment cycles (2 vs 4) (37).

In a large randomized trial, 290 women were assigned to a 6-month lifestyle-intervention program preceding 18 months of infertility treatment (intervention group) and 287 were assigned to prompt infertility treatment for 24 months (control group) (38). A discontinuation rate of 21.8% was documented in the intervention group. The average weight loss in the intervention group was 4.4 kg, with 43% reaching the target of 5% weight loss. At intention-to-treat analysis, the primary outcome, represented by the vaginal birth of a healthy singleton at term within 24 months after randomization, occurred in 27.1% of the women in the intervention group and 35.2% of those in the control group.

Despite these mixed results, it should be emphasized that weight loss before conception in women with obesity can reduce risks in pregnancy (39).

The effect of weight loss in obese women with PCOS was also evaluated in some studies. In a clinical trial, 149 women with excess weight and PCOS were randomized to 16 weeks of treatment with oral contraceptive pills (OCPs), lifestyle intervention with weight loss medication (sibutramine or orlistat), or to a combination of OCPs and lifestyle intervention (40). After preconception intervention, women underwent standardized ovulation induction with clomiphene citrate and timed intercourse for 4 cycles. A >6% weight loss was documented in both the lifestyle and the combined cohorts, associated with higher rates of ovulation compared with the OCP cohort. Although the study was not powered to detect a difference in LBR, a trend toward benefit with the use of the lifestyle intervention was documented (40).

In a secondary analysis of 2 randomized trials on infertile women with excess weight and with PCOS, immediate treatment with ovulation induction was compared with delayed treatment after a lifestyle intervention and weight loss (41). The analysis showed improved ovulation and live birth with delayed fertility therapy preceded by lifestyle modification compared with immediate therapy with clomiphene. Although the first-line therapy for PCOS is weight loss focusing on diet and regular exercise, it is well known that obese women with PCOS often fail to diet and disclose poor adherence to dietary behaviors. Thus, weight loss–targeted dietary modifications alone are usually not able to result in sustainable weight loss, with minimal results in metabolic and reproductive outcomes (42).

## Gut-Brain Axis Role in PCOS

Gut, brain, and metabolism are highly interrelated in obesity and DM, as well as in PCOS (43).

The central nervous system finely regulates food intake; however, even gastrointestinal hormones and other complex interactions influence eating behavior. Indeed, gut hormones and the gut-brain axis have several roles in the regulation of body fuel metabolism (44). Orexigenic and anorexigenic peptides mainly secreted in the gut produce short-term signals regarding the body energy status, while information on long-term energy stores is given by the serum levels of adipocyte-derived leptin (45).

The occurrence of obesity and insulin resistance in women with PCOS prompted investigation on underlying neuroendocrine interactions (46). In particular, the level of peripheral peptides in women with PCOS was extensively investigated. Serum levels of orexigenic peptides increase before the meals to promote eating activity and decrease after food intake (47). Ghrelin is the typical peripheral orexigenic peptide. Food consumption increases the levels of anorexigenic peptides such as cholecystokinin (CCK), to stop food intake, and GLP-1 and peptide YY (PYY), to regulate inter-meal periods (47).

Gastrointestinal peptides and adiposity signals regulate appetite through local and central effects (47).

Whether women with PCOS suffer from an alteration in appetite and satiety remains to be established, as does the role of gastrointestinal hormones in weight management.

In individuals with obesity, serum levels of ghrelin are lower than in lean individuals, and an increase in ghrelin levels is observed after weight loss (48-50). An increase in leptin and insulin levels has been blamed to explain the decrease in ghrelin levels in individuals with obesity; it has also been suggested that this alteration could represent a physiological adaptation of the body to obesity once a positive energy balance is reached (48, 49). Of note, ghrelin suppression, usually seen in lean individuals after meal consumption, is not found in individuals with obesity (48). The inability of food intake to suppress ghrelin levels can contribute to the pathogenesis of obesity.

Different results are available in the literature regarding ghrelin levels in women with PCOS. A meta-analysis of several studies suggests that fasting ghrelin levels are lower in PCOS; however, a significant heterogeneity across the included studies is documented (51). Available data also suggest either blunted suppression or no change of stimulated ghrelin levels in PCOS compared with BMI-matched healthy women. Ghrelin levels were found to be inversely correlated with parameters of hyperandrogenism, insulin levels, and insulin resistance (43).

Among gastrointestinal peptides, great attention has been devoted to GLP-1, a member of incretin hormones along with gastric inhibitory peptide (GIP) (52). Incretin hormones are secreted in the gut following food intake and they exert insulinotropic effects (52). GLP-1 is synthesized by L-cells in the distal small intestine and is secreted in response to food intake. The anorexigenic effects of GLP-1 are mediated by the vagus nerve, which provides communication between the gastrointestinal and the central nervous system (53, 54). Following gastric distension, the stimulation of mechanoreceptors generates satiety signals, which are conducted to brain via vagal nerves (55). GLP-1 slows down gastric emptying and intestinal motility both in healthy lean individuals and in subjects with obesity or type 2 DM (55).

The vagal afferents are, however, not the only entry of GLP-1 signaling to the central nervous system, as all blood–brain barrier-free areas of the central nervous system show high densities of GLP-1 receptors (56). Thus, activation of central pathways involved in appetite regulation by peripheral GLP-1 may occur via the vagus nerve, as well as directly via the area postrema and the median eminence (57). Evidence for a relationship between obesity and GLP-1 levels is mixed, with some studies showing decreased levels of GLP-1 (58-61) and others showing either increased basal GLP-1 or no significant change (62). Some studies also showed that meal-stimulated GLP-1 levels were lower in individuals with obesity when compared to lean subjects (63-65).

Secretion of GLP-1 after oral glucose tolerance test (OGTT) was evaluated in a group of lean, glucose-tolerant PCOS women in comparison with age- and BMI-matched healthy women (66). The study documented that active GLP-1 levels had a significantly different time-dependent pattern in PCOS (*P* < 0.002 for PCOS versus time interaction). GLP-1 concentrations were similar in PCOS and controls in the early phase of OGTT and then reached significantly lower levels in PCOS than in controls at 180  minutes (*P* < 0.05). These findings suggest that low late-phase active GLP-1 concentrations during OGTT could be used as an early marker of a prediabetic state in PCOS women.

The high prevalence of type 2 DM in PCOS gave birth to the hypothesis that GLP-1 secretion might be altered in this condition. However, women with obesity and PCOS apparently do not differ from controls with obesity in terms of basal and stimulated GLP-1 levels, although results are somewhat heterogeneous and inconclusive (43).

As for the role of other gastrointestinal peptides in women with PCOS, results are mixed about GIP secretion, while basal and meal-stimulated levels of CCK have been found to be similar in women with PCOS as compared with BMI-matched controls (67, 68). Similar basal and postprandial PYY levels in women with PCOS compared with BMI-matched healthy women have also been frequently reported (67-70).

### Endocrine and metabolic effects of GLP-1 RAs

Reduction in body weight has been demonstrated to improve hyperandrogenism, reproductive function, and metabolic parameters such as hyperlipidemia, and glycemic control, as well as hypertension, in women with PCOS (71-73). The availability of GLP-1 RAs offers a unique opportunity to simultaneously address both excess body weight and hyperglycemia. GLP-1 RAs are a class of glucose-lowering medications with incretin mimetic activity, approved for the treatment of type 2 DM. A full description of the mechanisms of action of GLP-1 RAs is out of the scope of this review and can be found elsewhere (74). In subjects with DM, the use of GLP-1 RA is associated with a significant reduction of glycated hemoglobin, with weight loss, a modest decrease in blood pressure, and an improvement of hyperlipidemia (75, 76). Recent cardiovascular outcomes trials have shown that some GLP-1 RAs, such as liraglutide and semaglutide, reduce the rate of major cardiovascular events in individuals with type 2 DM (77, 78).

Subcutaneous liraglutide 3 mg once daily is indicated as an adjunct to a reduced-energy diet and increased physical activity for chronic body weight management in adults with a BMI ≥30 kg/m^2^ or BMI of ≥27 kg/m^2^ and at least one weight-related comorbidity including hypertension, dyslipidemia, type 2 DM, or obstructive sleep apnea.

The efficacy of once-daily subcutaneous liraglutide injection for chronic weight management in adults was established in 5 large-scale randomized multicenter phase 3 trials (Table 1) (79-84). Four of these trials were part of the Satiety and Clinical Adiposity—Liraglutide Evidence in nondiabetic and diabetic individuals (SCALE) program.

**Table 1.** Characteristics of the RCTs Exploring the Role of Subcutaneous Liraglutide Injection for Chronic Weight Management in Adults

Study	Participant characteristics	Number randomized	Study duration	Results for liraglutide 3.0 mg vs placebo	Side effects for liraglutide 3.0 mg vs placebo	Attrition rate
Astrup A et al (79)	76% women stable body weight, BMI ≥30 kg/m^2^ and ≤40 kg/m^2^	Liraglutide 1.2 mg (N = 95) Liraglutide 1.8 mg (N = 90) Liraglutide 2.4 mg (N = 93) Liraglutide 3.0 mg (N = 93) Orlistat (N = 95) Placebo (N = 98)	20 weeks	Body weight: −4.4 kg (95% CI, −6.0 to −2.9 kg, *P <*0.0001); ≥5% body weight loss: 76.1% vs placebo 29.6% (*P <*0.0001); ≥10% body weight loss: 28.3% vs placebo 2.0%	Nausea (47.3% vs 5.1%) Diarrhea (12.9% vs 7.1%) Vomiting (11.8% vs 2.0%) Fatigue (10.8% vs 2.0%) Gastroenteritis (7.5% vs 3.1%)	16.3%
Astrup A et al (80) 2-year extension of (79)	76% women stable body weight, BMI ≥30 kg/m^2^ and ≤40 kg/m^2^	Liraglutide 1.2 mg (N = 95) Liraglutide 1.8 mg (N = 90) Liraglutide 2.4 mg (N = 93) Liraglutide 3.0 mg (N = 93) Orlistat (N = 95) Placebo (N = 98)	2 years	Body weight: −5.8 kg (95% CI, −8.0 to −3.7 kg, *P* *≤*0.001); ≥5% body weight loss: 73% vs placebo 28% (*P ≤*0.001); ≥10% body weight loss: 37% vs placebo 10% (*P ≤*0.001).	Nausea (48.4% vs 7.1%) Gastroenteritis (23.7% vs 13.3%) Influenza (23.7% vs 10.2%) Constipation (18.3% vs 12.2%) Diarrhea (15.1% vs 10.2%) URI (14.0% vs 9.2%) Fatigue (14.0% vs 8.2%) Vomiting (12.9% vs 2.0%)	23.8%
Wadden et al (81) SCALE Maintenance	81% women, stable body weight, BMI ≥30 kg/m^2^ or ≥27 kg/m^2^ with dyslipidemia or hypertension, loss ≥5 % of the initial body weight in low caloric diet run-in period (4-12 weeks)	Liraglutide 3.0 mg (N = 212) Placebo (N = 210)	56 weeks	Body weight: −5.9 kg (95% CI, −7.3 to −4.4 kg, *P <*0.0001); Body weight: −6.1 % (95% CI, −7.5 to −4.6%, *P <*0.0001); ≥5% body weight loss: 50.5% vs placebo 21.8% (*P <*0.0001); ≥10% body weight loss: 26.1% vs placebo 6.3% (*P <*0.0001); maintenance of ≥5% run-in weight loss: 81.4% vs placebo 48.9% (*P <*0.0001).	Nausea (47.6% vs 17.1%) Constipation (26.9% vs 12.4%) Diarrhea (17.9% vs 12.4%) Vomiting (16.5% vs 2.4%) Decreased appetite (9.9% vs 1.4%) Dyspepsia (9.4% vs 1.9%) Fatigue (8.0% vs 5.2%) Abdominal pain (6.6% vs 1.4%) Hypoglycemia (5.2% vs 2.4%)	27.7%
Pi-Sunyer et al (82) SCALE Obesity and prediabetes	78% women, stable body weight, BMI ≥30 kg/m^2^ or ≥27 kg/m^2^ with dyslipidemia or hypertension	Liraglutide 3.0 mg (N = 2487) Placebo (N = 1244)	56 weeks	Body weight: −5.6 kg (95% CI, −6.0 to −5.1 kg, *P <*0.0001); Body weight: −5.4% (95% CI, −5.8 to −5.0%, *P <*0.001); ≥5% body weight loss: 63.2% vs placebo 27.1% (*P <*0.001); ≥10% body weight loss: 33.1% vs placebo 10.6% (*P <*0.001).	Nausea (40.2% vs 14.7%) Diarrhea (20.9% vs 9.3%) Constipation (20.0% vs 8.7%) Vomiting (16.3% vs 4.1%) Decreased appetite (10.8% vs 3.1%) Dyspepsia (9.5% vs 3.1%)	30.6%
Davies et al (83) SCALE Diabetes	50% women, stable body weight, BMI ≥27 kg/m^2^, type 2 diabetes (HbA1c 7.0%-10.0%) treated with diet and exercise alone or in combination with 1-3 oral glucose- lowering agents	Liraglutide 3.0 mg (N = 423) Liraglutide 1.8 mg (N = 211) Placebo (N = 212)	56 weeks	Body weight: −4.2 kg; Body weight: −4.0 % (95% CI, −5.1 to −2.9%, *P <*0.001); ≥5% body weight loss: 54.3% vs placebo 21.4% (*P <*0.001); ≥10% body weight loss: 25.2% vs placebo 6.7% (*P <*0.001).	Hypoglycemia^a^ (44.5% vs 27.4%) Nausea (32.7% vs 13.7%) Diarrhea (25.6% vs 12.7%) Constipation (16.1% vs 6.1%) Vomiting (15.6% vs 5.7%) Dyspepsia (11.1% vs 2.4%) Abdominal distension (6.2% vs 1.4%) Abdominal pain (6.2% vs 4.2%)	25.8%
Blackman et al (84) SCALE Sleep Apnea	28% women, stable body weight, BMI ≥30 kg/m^2^, moderate to severe OSA, unwilling or unable to use CPAP	Liraglutide 3.0 mg (N = 180) Placebo (N = 179)	32 weeks	Body weight: −4.9 kg (95% CI, −6.2 to −3.7 kg, *P <* 0.0001); Body weight: −4.2 % (95% CI, −5.2 to −3.1%, *P <*0.0001); ≥5% body weight loss: 46.4% vs placebo 18.1% (*P <*0.0001); ≥10% body weight loss: 22.4% vs placebo 1.5% (*P <*0.0001).	Nausea (26.7% vs 6.7%) Diarrhea (16.5% vs 7.8%) Constipation (11.9% vs 3.4%) Dyspepsia (8.5% vs0.1.1%) Vomiting (7.4% vs 2.8%) GERD (5.7% vs 0.6%)	23.1%

^a^American Diabetes Association definition.

Abbreviations: BMI, body mass index; CPAP, continuous positive airway pressure; GERD, gastroesophageal reflux disease OSA, obstructive sleep apnea; RCT, randomized controlled trial; URI, upper respiratory tract infection.

In a double-blind, placebo-controlled, 20-week trial, with open-label orlistat comparator, 564 individuals were randomly assigned to 1 of 4 liraglutide doses (1.2 mg, 1.8 mg, 2.4 mg, or 3.0 mg) or to placebo administered once a day subcutaneously, or orlistat (120 mg) 3 times a day orally (79). Mean weight loss with liraglutide 1.2 to 3.0 mg was 4.8 kg, 5.5 kg, 6.3 kg, and 7.2 kg compared with 2.8 kg with placebo and 4.1 kg with orlistat. More individuals (76%) lost more than 5% weight with liraglutide 3.0 mg than with placebo (30%) or orlistat (44%). Liraglutide reduced blood pressure at all doses, and reduced the prevalence of prediabetes (84%-96% reduction) with 1.8 to 3.0 mg per day (79). The benefits of liraglutide on body weight and cardiovascular risk factors were maintained over 2 years (80).

In a phase 3 randomized trial, participants with overweight/obesity who lost ≥ 5% of initial weight during a low-calorie diet run-in were randomly assigned to liraglutide 3.0 mg per day or placebo for 56 weeks (81). Participants lost a mean 6.0% of their weight during run-in. From randomization to week 56, weight decreased an additional mean 6.2% with liraglutide and 0.2% with placebo. More participants receiving liraglutide (81.4%) maintained the ≥5% run-in weight loss, compared with those receiving placebo (48.9%), and 50.5% vs 21.8% of participants lost ≥5% of randomization weight.

A large 56-week, double-blind trial involved 3731 patients without type 2 DM and with BMI ≥30 or ≥27 if they had treated or untreated dyslipidemia or hypertension (82). Patients were randomly assigned in a 2:1 ratio to receive once-daily subcutaneous injections of 3.0 mg liraglutide or placebo. At week 56, patients in the liraglutide group had lost a mean of 8.4 ± 7.3 kg of body weight, and those in the placebo group had lost a mean of 2.8 ± 6.5 kg. A total of 63.2% of the patients in the liraglutide group, as compared with 27.1% in the placebo group, lost at least 5% of their body weight, moreover 33.1% and 10.6%, respectively, lost more than 10% of their body weight.

In a 56 week double-blind, placebo-controlled trial, 846 patients with overweight or obesity and type 2 DM were randomized in a 2:1:1 ratio to liraglutide 3 mg, liraglutide 1.8 mg, or placebo, all as adjunct to 500 kcal/day intake less than required for weight maintenance and increased physical activity (≥150 min/wk) (83). Weight loss was 6.0% (6.4 kg) with liraglutide (3.0 mg dose), 4.7% (5.0 kg) with liraglutide (1.8 mg dose), and 2.0% (2.2 kg) with placebo. Weight loss of ≥5% occurred in 54.3% with liraglutide 3.0 mg and 40.4% with liraglutide 1.8 mg versus 21.4% with placebo. Weight loss >10% occurred in 25.2% with liraglutide 3.0 mg and 15.9% with liraglutide 1.8 mg versus 6.7% with placebo.

Finally, a 32-week, double-blind, randomized trial investigated whether liraglutide 3.0 mg reduced obstructive sleep apnea (OSA) severity compared with placebo in individuals with obesity and without diabetes (84). The study documented a significant reduction in OSA severity, as measured with the apnea-hypopnea index (AHI), in the liraglutide group versus placebo. Liraglutide produced greater mean percentage weight loss compared with placebo (−5.7% vs −1.6%). A statistically significant association between the degree of weight loss and improvement in OSA endpoints was also documented.

A recent meta-analysis of these trials showed that treatment with liraglutide produced an average decrease in body weight of 5.25 kg (95% CI, −6.17 to −4.32), compared with placebo (85).

Recently, the use of GLP-1 RAs has been extended to other conditions such as PCOS.

## Liraglutide in PCOS

### Effects of GLP1-RA on metabolic endpoints

Several studies have shown that short-term treatment with GLP-1 RAs, either as monotherapy or in combination with metformin, produces significant weight loss and favorable metabolic changes in women with overweight or obesity and PCOS. Clinical studies of GLP-1 RAs in obese/overweight PCOS women are summarized in Table 2. Most of the evidence refers to the use of liraglutide in patients previously treated with metformin. In addition to a relevant effect on weight, some studies documented additional beneficial effects on androgen levels and metabolic parameters (86, 87).

**Table 2.** Clinical Studies of GLP-1 RAs in Obese/Overweight Women With PCOS

Study	Participant characteristics	Study arms	Study duration	Primary outcome	Other outcomes	Body weight Loss (kg)	Metabolic changes	Menstrual pattern/other	Attrition rate
Elkind- Hirsch et al (86)	Overweight (BMI >27), insulin-resistant, oligoovulatory women. Age 18-40 years. PCOS diagnosed according to a modification of Rotterdam criteria 2003	1) MET:1000 mg bid (n = 14) 2) EXE (10 μg bid) (n = 14) 3) MET 1000 mg bid + EX 10 μg bid (n = 14)	24 weeks	Menstrual frequency	Ovulation rate Insulin action FAI Inflammatory markers	1) −1.6 ± 0.2 2) −3.2 ± 0.1 3) −6.0 ± 0.5	FAI reduced and Insulin sensitivity improved in combination arm	Combination therapy superior to EX or MET (menses and ovulation)	30%
Rasmussen et al (87)	Overweight or obese women, pre-treated with MET for at least 6 months. PCOS diagnosed according to Rotterdam criteria.	Add-on of LIRA 1.2-1.8 mg (n = 84)	Mean of 27.8 weeks	Weight loss	-	−9.0 (95% CI, 7.8- 10.1)	Mean decrease in BMI of 3.2 kg/m^2^ Weight loss >5% and >10% in 81.7% and 32.9% of patients, respectively		20.0%
Kahal et al (88)	Obese women (BMI 30-45) Age 18-45 years Healthy controls. PCOS diagnosed according to Rotterdam criteria.	LIRA:1.8 mg (19 obese women with PCOS and 17 controls)	6 months	CV risk markers	Weight loss	PCOS: -3.0 ± 4.2 Controls: −3.8 ± 3.0	HOMA-IR, hsCRP, endothelial adhesion markers significantly and equally reduced in both groups No changes in cIMT		30.6%
Jensterle et al (89)	Obese (BMI ≥30) women Age ≥18 years, premenopause. PCOS diagnosed by ASRM-ESHRE Rotterdam criteria.	1) MET:1000 mg bid (n = 14) 2) LIRA 1.2 mg (n = 14) 3) ROF 500 mg qd (n = 14)	12 weeks	Weight loss	Hormonal and metabolic changes	1) −0.8 ± 1.0 2) −3.1 ± 3.5 3) −2.1 ± 2.0	FAI reduction in ROF arm; decrease in visceral fat in LIRA arm. HOMA-IR decreased in all treatment arms. LIRA superior to MET in reducing glucose at 120 min of OGTT	Menstrual frequency increased with all treatments.	8.9%
Frøssing et al (90)	Women with BMI >25 and/ or insulin resistance PCOS diagnosed according to Rotterdam criteria	1) LIRA 1.8 mg OD s.c. (n = 48) 2) Placebo (n = 24)	26 weeks	Liver fat VAT Prevalence of NAFLD	Weight change OGTT, SHBG, testosterone	1) −5.2 ± 0.7 2) +0.2 ± 0.9	LIRA reduced liver fat content by 44%, VAT by 18%, and the prevalence of NAFLD by two-thirds. With LIRA, SHBG levels increased by 19%, and free testosterone decreased by 19%.		28%
Jensterle Sever et al (91)	Obese (BMI ≥30), nondiabetic women who had lost ≥5% body weight during pretreatment with MET for at least 6 months. Age >18 years. PCOS diagnosed by the NICHD criteria.	1) MET: 1000 mg BID (n = 14) 2) LIRA: 1.2 mg OD s.c. (n = 11) 3) MET 1000 mg BID + LIRA 1.2 mg QD s.c.(n = 11)	12 weeks	Weight loss	Body composition IR	1) −1.2 ± 1.4 2) −3.8 ± 3.7 3) −6.5 ± 2.8	No major changes in fasting glucose, insulin, and insulin during OGTT. Glucose value after 120 min during OGTT significantly reduced in the combination arm vs MET.	No significant changes in menstrual pattern.	10%
Jensterle et al (92)	Obese (BMI ≥30) women with newly diagnosed PCOS. Age ≥18 years, premenopause. PCOS diagnosed by the NICHD criteria.	1) MET:1000 mg bid/ daily (n = 14) 2) LIRA 1.2 mg (n = 14)	12 weeks	Weight loss	Body composition IR	1) −2.3 2) −3.0 LIRA superior in severe IR (BMI reduction 2.13 vs 0.62 kg/m^2^)	Decrease in total testosterone in MET Increase in LH in LIRA		12.5%
Jensterle et al (94)	Obese (BMI ≥30) women Age ≥18 years, premenopause PCOS diagnosed by ASRM-ESHRE Rotterdam criteria	1) MET 1000 mg BID + LIRA 1.3 mg OD s.c. (n = 15) 2) LIRA 3 mg OD s.c. (n = 15)	12 weeks	Weight loss	Metabolic and hormonal changes	1) −3.6 ± 2.5 2) −6.3 ± 3.7	Both interventions resulted in a significant decrease of post-OGTT glucose levels. Combination therapy significantly reduced total testosterone		6.7%
Nylander et al (96).	Overweight (BMI ≥25) women and/ or insulin resistance Age ≥18 years PCOS diagnosed according to Rotterdam criteria	1) LIRA 1.8 mg (n = 44) 2) Placebo (n = 21)	26 weeks	Bleeding pattern	Levels of AMH, sex hormones, and gonadotrophins, ovarian morphology	LIRA vs Placebo: −5.2 (95% CI, 3.0-7.5)	In the LIRA group, SHBG increased and free testosterone decreased. Ovarian volume decreased with LIRA vs placebo.	Bleeding ratio significantly improved with LIRA vs placebo	9.7%
Salamun et al (97)	Infertile, obese women (BMI ≥30) Age ≤38 years, first or second IVF attempt PCOS diagnosed according to revised Rotterdam criteria	1) MET 1000 mg bid (n = 14) 2) MET 1000 mg bid + LIRA 1.3 mg QD s.c. (n = 14)	12 weeks	IVF pregnancy rate	Weight change	1) −7.0 ± 6.0 2) −7.5 ± 3.9		Pregnancy rate per embryo transfer significantly higher with combined therapy (85.7%) compared with MET (28.6%)	18%
Liu et al (98).	Overweight or obese women (BMI ≥24) Age 18-40 years PCOS diagnosed according to Rotterdam criteria	1) MET:1000 mg bid (n = 88) 2) EXE: 10 μg BID (n = 88)	12 weeks; after 12 weeks all patients treated with MET alone for additional 12 weeks	Weight loss	Fat mass, hormonal levels, insulin resistance, lipid profile, inflammatory marker, menstrual frequency, and rate of pregnancy.	1) −2.28 ± 0.55 2) −4.29 ± 1.29	Decrease in android fat mass% and total fat mass% with EXE, but not with MET. Greater decrease in HOMA-IT and insulin levels with EXE than with MET. HsCRP levels decreased significantly in the EXE group only.	Menstrual frequency increased significantly in both groups. Rate of natural pregnancy significantly higher following EXE treatment than following MET treatment	10.2%

AMH, anti-Müllerian hormone; cIMT, carotid-intima media thickness; EXE, exenatide; FAI, free androgen index; HOMA-IR, homeostasis model assessment-IR; hsCRP, high-sensitivity C-reactive protein; IR, insulin resistance; IVF, in vitro fertilization; LIRA, liraglutide; MET, metformin; NAFLD, nonalcoholic fatty liver disease; NICHD, National Institute of Child Health and Human Development; OGTT, oral glucose tolerance test; PIIINP, Procollagen Type III N-Terminal Peptide; SHBG, sex hormone binding globulin; VAT, visceral adipose tissue.

In a 6-month controlled trial, the efficacy of daily liraglutide 1.8 mg on weight loss and markers of atherothrombosis was assessed in 19 women with obesity and PCOS and 17 controls of similar age and weight. In both groups, liraglutide treatment was associated with weight loss of 3% to 4% and significant reduction in atherothrombosis markers, including inflammation, endothelial function, and platelet function (88).

In a 12-week randomized study, 45 women with obesity and PCOS were allocated to metformin 1000 mg twice daily or liraglutide 1.2 mg daily or roflumilast 500 μg daily (89). Significantly greater BMI reductions were documented with liraglutide or roflumilast (1.1 ± 1.26 and 0.8 ± 0.99 kg/m^2^, respectively) as compared with metformin (0.1 ± 0.67 kg/m^2^). The use of liraglutide was also associated with improvements in body composition, as assessed by dual energy x-ray absorptiometry (DXA), including a significant decrease of visceral adipose tissue area.

The effect of liraglutide on ectopic fat was further evaluated in a double-blind, placebo-controlled, randomized clinical trial involving 72 women with PCOS (90). Compared with placebo, 26 weeks of treatment with liraglutide significantly reduced body weight by 5.2 kg, liver fat content by 44%, visceral adipose depot by 18%, and the prevalence of nonalcoholic fatty liver disease by two-thirds.

The results of 7 randomized trials assessing the efficacy of liraglutide in women with PCOS (86-89, 91, 92) have been combined in a meta-analysis (93). The outcomes assessed included BMI and waist circumference, fasting serum insulin levels and insulin resistance as assessed by HOMA-IR, and androgenic status including serum total testosterone and sex hormone-binding globulin (SHBG) concentrations. Liraglutide treatment decreased BMI by 1.65 (0.72-2.58) kg/m^2^ in 172 patients after 3 months. In addition, a decrease in serum testosterone levels was observed, while insulin levels and insulin sensitivity were not reduced, but had high heterogeneity among studies.

Short-term interventions with metformin plus liraglutide 1.2 mg (COMBO) or liraglutide 3.0 mg (LIRA 3 mg) alone led to significant weight loss (−3.6 ± 2.5 kg, *P* = 0.002 in COMBO vs −6.3 ± 3.7 kg, *P* = 0.001 in LIRA3) in obese women with PCOS; liraglutide 3.0 mg was superior to liraglutide 1.2 mg plus metformin in BMI and waist circumference (WC) reduction (BMI: LIRA 3 − 2.2 ± 1.3 vs COMBO − 1.3 ± 0.9 kg/m^2^, *P* = 0.05; WC: LIRA3 − 4.2 ± 3.4 vs COMBO − 2.2 ± 6.2 cm, *P* = 0.014) (94).

More recently, a meta-analysis of 8 randomized trials compared the efficacy of GLP-1 RAs versus metformin in women with PCOS (95). A GLP-1 RA was superior to metformin in improving insulin sensitivity and reducing BMI and abdominal circumference. GLP-1 RAs had similar effects on menstrual frequency, serum total testosterone, free androgen index, SHBG, dehydroepiandrosterone sulfate (DHEA-S), Ferriman-Gallwey scores, androstenedione, LH, fasting blood glucose, fasting insulin, triglycerides, total cholesterol, and blood pressure when compared with metformin.

### Effects of GLP1-RA on reproductive endpoints

The effects of liraglutide on ovarian dysfunction in PCOS were evaluated in a double-blind, randomized trial (96). In this study, 72 women with PCOS were allocated to intervention with liraglutide or placebo (1.8 mg/day), in a 2:1 ratio. Bleeding pattern, levels of anti-Müllerian hormone (AMH), sex hormones, and gonadotrophins were assessed and ovarian morphology evaluated. After 26 weeks, liraglutide significantly reduced weight loss by an average of 5.2 kg compared with placebo. The use of liraglutide was also associated with an improvement in bleeding ratio, an increase in SHBG, and a decrease in free testosterone and ovarian volume versus placebo. Contrary to these results, other studies with liraglutide in PCOS found unchanged bleeding frequency despite a decrease in body weight (89-96) and insulin resistance (88). Small sample size, short duration of treatment, and suboptimal liraglutide dose can represent likely explanations for these negative findings.

The impact of liraglutide treatment on pregnancy rates has been seldom investigated. In a randomized, open-label pilot study on 28 women with obesity and PCOS, a 12-week preconception treatment with low-dose liraglutide (1.2 mg daily) in combination with metformin was superior to metformin alone in increasing in vitro fertilization pregnancy rates (97). An increase in the cumulative pregnancy rate was also documented, including spontaneous pregnancies after treatment in women who had been previously been resistant to lifestyle modification and first-line reproductive treatment. Pregnancy rate per embryo transfer was significantly higher in the liraglutide plus metformin group compared to metformin alone (85.7% vs 28.6%, respectively). The cumulative pregnancy rate in a time-frame of 12 months was 69% with the combination therapy compared with 36% in the metformin group.

Natural pregnancy rate was investigated in a clinical trial involving 176 overweight or obese women with PCOS, who were randomized to receive either exenatide 10 μg twice daily or metformin 1000 mg twice daily for the first 12 weeks. Then all patients were treated with metformin alone during the second 12 weeks (98). During the second 12 weeks, the rate of natural pregnancy of exenatide-treated patients was significantly higher than metformin-treated patients (43.60% vs 18.70%; *P* < 0.05).

## Conclusions

The dramatic increase in obesity and overweight prevalence and its long-term health sequelae are associated with a decline in fertility rates throughout the developed world. The association between excess weight and poor fertility outcomes is indisputable, and weight reduction represents the most significant factor affecting fertility and pregnancy outcomes.

Current experimental and clinical evidence suggests the presence of an underlying pathophysiological link between obesity, GLP-1 kinetic alterations, and PCOS pathogenesis.

Based on the positive results in patients affected by obesity, with or without diabetes, the administration of GLP-1 RA (mainly liraglutide) alone or in combination with metformin has been investigated in women with obesity and PCOS. Several studies demonstrated significant weight loss and testosterone reduction, with mixed results relative to improvements in insulin resistance parameters and menstrual patterns. That being so, the weight loss effects of GLP-1 RA offer a unique opportunity to expand the treatment options available to PCOS patients. This may be extremely useful under some circumstances, such as in assisted reproductive settings when women seeking help for infertility have advanced age and/or poor ovarian reserve. Moreover, these women, being overweight/obese, carry higher risks during controlled ovarian stimulation and throughout pregnancy, which can be minimized by a pretreatment with the specific goal to significantly reduce body weight.

In conclusion, the weight loss effects of GLP-1 RA offer a unique opportunity to expand the treatment options available to PCOS patients. Treatments should be part of a comprehensive, multidisciplinary approach for weight management in women with overweight/obesity and PCOS, and especially in those planning to conceive following assisted reproductive technology. At present, the best strategy starts with comprehensive lifestyle management and may include the use of approved weight loss medications to ameliorate comorbidities and achieve meaningful clinical outcomes.

## Abbreviations

BMI: body mass index
CCK: cholecystokinin
DM: diabetes mellitus
GLP-1: glucagon-like peptide-1
GLP-1 RA: glucagon-like peptide-1 receptor agonist
HPO: hypothalamic-pituitary-ovarian
IVF: in vitro fertilization
LBR: live birth rate
LH: luteinizing hormone
OCP: oral contraceptive pills
OSA: obstructive sleep apnea
PCOS: polycystic ovary syndrome
PYY: peptide YY
SHBG: sex hormone–binding globulin

## Additional Information

***Disclosure Summary*:** The authors have no conflict of interest to disclose.

***Data Availability:*** Data sharing is not applicable to this article as no datasets were generated or analyzed during the current study.
